# In-stent Stenosis after p64 Flow Diverter Treatment

**DOI:** 10.1007/s00062-017-0591-y

**Published:** 2017-05-09

**Authors:** M. Aguilar Pérez, P. Bhogal, E. Henkes, O. Ganslandt, H. Bäzner, H. Henkes

**Affiliations:** 1 Neuroradiological Clinic, Neurocenter, Klinikum Stuttgart, Kriegsbergstrasse 60, 70174 Stuttgart, Germany; 2 Neurosurgical Clinic, Neurocenter, Klinikum Stuttgart, Stuttgart, Germany; 3 Neurological Clinic, Neurocenter, Klinikum Stuttgart, Stuttgart, Germany; 4 Medical Faculty, University Duisburg-Essen, Essen, Germany

**Keywords:** Flow diverter, Stent, Aneurysm, In stent stenosis, p64

## Abstract

**Purpose:**

There is limited information available on the incidence of in-stent stenosis (ISS) secondary to the use of flow-diverting stents in the intracranial vasculature. We sought to determine the incidence, severity, and clinical course of ISS on angiographic follow-up after treatment of saccular aneurysms with p64.

**Methods:**

We retrospectively reviewed all patients who underwent treatment of a saccular (ruptured and unruptured) intracranial aneurysm with ≥1 p64 between 2011 and 2015. Fusiform aneurysms and dissections were excluded. Aneurysms with prior or concomitant saccular treatment (e. g., coiling, clipping) were included. Extradural targets and aneurysms with parent vessel implants other than p64 were excluded. ISS was assessed on follow-up angiography and defined as <50% (mild), 50–75% (moderate), or >75% (severe).

**Results:**

In total, 205 patients (147 female, 71.7%; median age 57 years), with 223 saccular aneurysms were treated with p64 and had at least 9 months of digital subtraction angiography (DSA) follow-up completed. There was no DSA follow-up available in 8 patients. ISS of any degree at any time was recognized in 65/223 (29.1%) of all target aneurysms. The maximal degree of lumen loss was <50% in 40 lesions (17.9%), 50–75% in 19 lesions (8.5%), and >75% in 6 lesions (2.7%). ISS did not cause a focal neurological deficit in any patient. No progression from stenosis to occlusion was observed. Balloon angioplasty was performed in 1 lesion and was well tolerated. In 56 lesions (84.8%), a significant reduction of ISS occurred spontaneously, 2 mild stenoses remained stable, and for 6 lesions the long-term follow-up is pending.

**Conclusion:**

Treatment with p64 is associated with an overall rate of 8.5% moderate ISS (50–75%) and 2.7% severe ISS (>75%), which is comparable with the rate of ISS reported in the literature for other flow diverting stents. There is a tendency for ISS to spontaneously improve over time.

## Introduction

The recent introduction of flow-diverting stents (FDS) into clinical practice to treat intracranial vascular disease has revolutionized the treatment of intracranial aneurysms. While much attention has been paid to aneurysm cure rates, ischemic complications and hemorrhage, little data has been published on the incidence of in-stent stenosis (ISS). The p64 is a flow-diverting implant which differs in material (nitinol and platinum), porosity (51–60%), and radial force from other devices with the same intended use. This may have an effect on the incidence and course of ISS. It was our aim to determine the incidence, clinical significance, treatment, and course of ISS associated with p64 used for the treatment of saccular aneurysms.

## Methods

### Patient Population

Between 14 December 2011 and 31 December 2015, 233 patients with 255 intracranial saccular aneurysms without previous treatment of the parent vessel were admitted to our institution for endovascular treatment with the p64 flow diverter. For each patient we recorded demographic data, clinical presentation, location of the target lesion, therapeutic intervention, immediate angiographic and clinical result, and for 205 patients with 223 aneurysms clinical and radiological follow-up information were available. The data was anonymously entered into a computer database.

### Grading of In-stent Stenosis

ISS was defined as any lumen loss within the implanted p64(s). Angiographically this appears as a gap between the contrast filled vessel lumen and the inner contour of the p64. When there was no such appearance, there was no evidence of angiographic ISS. ISS was graded to be <50% (mild), 50–75% (moderate), or >75% (severe). Measurements were carried out using the PACS software (AGFA), which yields noncalibrated relative distances.

### Endovascular Treatment

All treatments were performed under general anesthesia. Patient informed consent was obtained before the procedure in all cases. At least one p64 was used in all patients.

All patients received dual antiplatelet medication with either 1 × 100 mg ASA (aspirin) per oral daily and 1 × 75 mg clopidogrel per oral daily for at least 3 days or with 1 × 500 mg ASA and 1 × 600 mg clopidogrel 24 h prior to the treatment. The effectiveness of the antiplatelet regime was tested using the Multiplate® Analyzer (Roche) and in case of doubt with the VerifyNow® System (Accriva). Patients nonresponsive to clopidogrel received either 1 × 10 mg prasugrel or 2 × 90 mg ticagrelor per oral daily. No p64 was implanted unless a significant platelet inhibition was confirmed. The postprocedural regimen included dual antiplatelet medication for 12 months, followed by 100 mg ASA per oral daily continued for life.

Standard procedures were performed via the right common femoral route using a 6 F sheath and guide catheter. All procedures were performed under heparin anticoagulation with a 3000 IU bolus dose at the start of the procedure and subsequent 1000 IU bolus doses every hour to maintain the activated clotting time between 2–2.5 times the baseline.

### Procedural Assessment and DSA Follow-up Evaluation

Patency and flow characteristics within the flow diverter and distal branches were assessed angiographically immediately after placement of the p64s and during follow-up. Follow-up DSAs were scheduled at 3, 9, and 24 months after the treatment. In the case of ISS or other potential issues, additional follow-up DSA examinations were offered according to individual clinical requirements. Only patients with at least 9 months DSA follow-up were analyzed. Standard angiographic projections were used to assess the patency of the vessels and the aneurysms in addition to the angiographic projections that repeated those used during the treatment.

Neurological examinations were performed to evaluate for potential ischemic or hemorrhagic complications in the postoperative period (<24 h post procedure) and at each subsequent follow-up visit.

If ISS was seen on any study, the antiaggregation effect was tested using both the Mutliplate and Verify Now systems to ensure an adequate level of platelet inhibition. Once an adequate level of platelet inhibition was ensured, dual antiplatelet medication was continued until the ISS had resolved.

## Results

A total of 233 patients (165 female, median age 57 years) were treated for saccular aneurysms with at least one p64 and 205/233 (88.0%) had follow-up DSA examinations per clinical protocol for at least 9 months, making them eligible for inclusion in this study. A total of 255 aneurysms were treated with p64 and 223/255 (87.4%) had at least 9 months DSA follow-up. Six patients were treated for ruptured aneurysms and the remaining 227 patients had unruptured aneurysms. A total of 240 aneurysms in 219 patients were covered by one p64 implanted, in 12 cases two p64 were implanted, and in 3 patients three p64 were implanted.

In total, 23 complications occurred (9%): 6 (2.4%) in the periprocedural period (<24 h), 7 (2.7%) in the postprocedural phase (24 h–30 days), and 10 (3.9%) occurred after 30 days. The majority of these complications were ischemic in nature including all seven that occurred in the postprocedural period and six of those that occurred after 30 days. Two patients had subarachnoid hemorrhages (one postprocedural contralateral asymptomatic, one traumatic during the F/U period). There were three deaths following the procedure in this cohort: 1 patient died from a traumatic ICH during the follow-up period, 1 patient died from pneumonia due to excessive steroid consumption, and 1 died unrelated to the treatment.

In all, 205 patients, with 223 aneurysms, were available for a DSA follow-up of at least 9 months or longer. Of the 223 aneurysms, 158 were associated with no ISS (70.8%), 40 aneurysms (17.9%) were associated with mild ISS (<50%), 19 aneurysms (8.5%) were associated with moderate ISS (50–75%), and 6 (2.7%) were associated with severe stenosis (>75%).

**Fig. 1:**
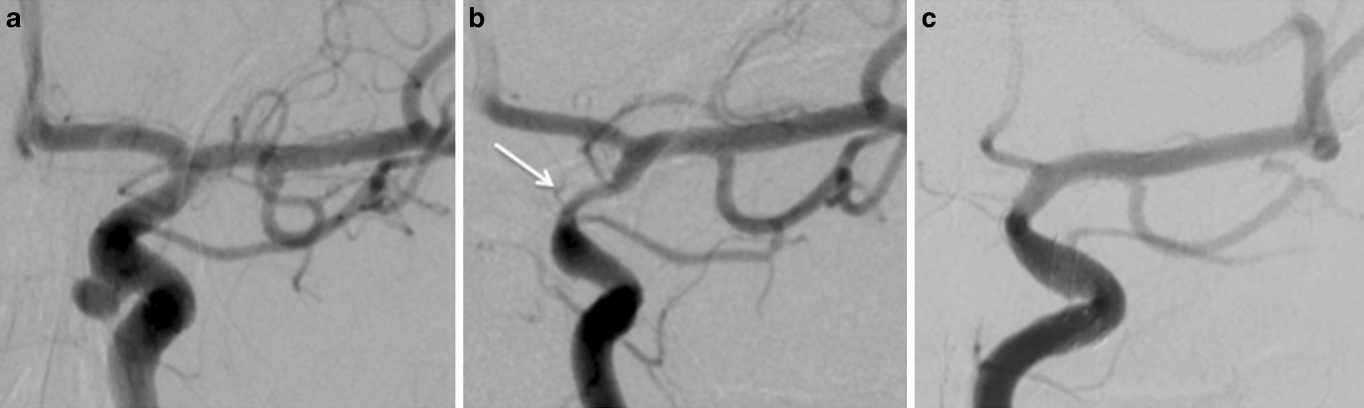
A patient treated with a single p64 flow diverter for an aneurysm related to the superior hypophyseal artery (**a**). A follow-up angiogram at 4 months (**b**) showed severe in-stent stenosis (*arrow*). She underwent balloon angioplasty. A delayed angiogram 12 months after this balloon angioplasty shows only mild residual stenosis with no flow limitation

Of the 149 patients with 163 lesions with no or insignificant stenosis on the initial follow-up angiogram (3 months), 3 patients went on to develop mild stenosis and two developed moderate stenosis. Of those 39 lesions with mild stenosis on the initial angiogram (3 months), 28 had complete resolution and 9 lesions remained stable on the mid-term follow-up and only two persisted and further six were resolved on the long-term follow-up which is pending for 1 patient. Two patients developed worsening (one to moderate, one to high grade) ISS at the subsequent DSA (9 months). Of those 16 patients with moderate stenosis on the 3 months angiogram, 12 had either complete resolution or improvement of their stenosis with 4 patients remaining stable and only one was not improved on the 1‑year follow-up. All 5 of the patients with severe stenosis at 3 months had delayed angiograms, 1 was treated with balloon dilatation early in our experience (Fig. 1) and four had either improved or resolved at follow-up imaging.

Overall the degree of any ISS was seen in 66 (29.6%) of 223 aneurysms at subsequent angiographies and there was a trend for this to improve spontaneously over time.

Of the 13 patients who had multiple p64 stents implanted one showed ISS stenosis, which resolved spontaneously. There was no in-stent stenosis associated with the 3 ruptured aneurysms available for the 9 months follow-up.

In 3 patients (1.5%) an occlusion of the p64 was encountered. This was in no case associated with a known ISS and in all patients related to inconsistencies with their dual antiplatelet medication.

## Discussion

Since their introduction into clinical practice flow diverters have provided a viable alternative treatment strategy for complex aneurysms previously not amenable to endovascular management. Numerous studies have shown a favorable safety and efficacy profile [1–5]. The recent meta-analysis performed by Brinjikji et al. [6] found procedure-related morbidity and mortality of 5 and 4%, respectively. The meta-analysis of Wang et al. [7] focused exclusively on the use of flow diverters in the posterior circulation. In this scenario procedure related mortality was 15% and ischemic stroke rate 11%.

While this information is no doubt of extreme importance when determining appropriate treatment strategies and consulting patients, little has been published on intimal hyperplasia and in-stent stenosis following the use of flow diverters in the intracranial vasculature.

Cohen et al. [8] reviewed the delayed consequences of flow diverters used to treat intracranial ICA aneurysms <25 mm in diameter in 34 patients, 18 treated with the PED and 16 treated with the Silk (Balt, Montmercy, France) flow diverters. Serial angiography was performed at 2, 6, 9 to 12, and 16–20 months post procedure. In-stent stenosis was defined as any growth process beyond the strict limits of the metallic mesh of the stent metallic mesh and was graded as mild (<25%), moderate (25–50%), or severe (>50%). In-stent stenosis was detected in 38% of Silk flow diverters and 39% of PED’s on initial follow-up angiography. This, in the majority of cases was mild (9/13); however, 1 patient had moderate stenosis and another severe, both of which were seen in association with the Silk stent. One patient was symptomatic and suffered from recurrent TIA symptoms; however, in addition to the mild in-stent stenosis, the patient also had severe distal tapering of the Silk and it is more likely the latter was the cause of the clinical symptoms.

In the more recent publication of John et al. [9], long-term angiographic follow-up (mean length 12.5 months) was available in 51 patients treated with the PED. In this retrospective study intimal hyperplasia was defined as 1–25% narrowing. In-stent stenosis was defined as mild (25–50%), moderate (50–75%), or severe (>75%). In-stent stenosis was seen in 5 patients (>25 narrowing) and in 4 patients was graded as mild and the fifth patient moderate. None of these patients had flow limitation or clinical symptoms. However, a further 16 patients had intimal hyperplasia (31%). According to the definition of <25% narrowing as intimal hyperplasia, we can see that 26% of patients had intimal hyperplasia in the study of Cohen et al. (9/34). Similarly, the overall rate of any in-stent stenosis (excluding complete occlusion) in the study by John et al. is 41%, which is remarkably similar to that seen in the Cohen et al. cohort (overall 38%). The findings of the study conducted by Lubicz et al. [10] are more difficult to directly compare principally because of the difference in definition (<50% mild, >50% severe). However, in this study 22 patients had mild stenosis (49%) which is considerably higher than that reported by the previously mentioned groups. Our findings of an overall incidence of 29% in-stent stenosis and 32% of patients treated are congruent with the findings of other authors.

The cellular responses that occur following mechanical vascular injury immediately after stent deployment can be divided into three different phases [11–13]:

- Early phase – platelet activation and inflammation
- Intermediate phase – granulation tissue and smooth muscle cell migration and proliferation
- Late phase – tissue remodeling

In the early phase endothelial cells are partially or completely destroyed or crushed and this causes the activation and aggregation of platelets, infiltrating leucocytes and the release of various growth factors and cytokines [11, 14, 15]. The response of the endothelium to stent-induced damage can be further subdivided into three phases – endothelial denudation, re-endothelialization, and/or neo-endothelialization. The initial destruction of the endothelium initiates the formation of a thin thrombus layer that covers the vascular and stent surface even in the presence of dual antiplatelet agents and heparin [12]. Subsequent to platelet activation and aggregation there is recruitment of circulating leucocytes [15]. This leucocyte–platelet interaction is critical in the initiation and progression of neointimal formation [16].

During the intermediate phase, endothelial cells proliferate and migrate over the injured areas with vascular smooth muscle cells (VSMCs) and macrophages replacing the fibrin clot with granulation tissue. The macrophages enhance the inflammatory response and secrete numerous chemokines and growth factors and these are required for the healing of the wounded endothelial surface.

During the late tissue remodeling phase, there is modification of the VSMCs from contractile to synthetic phenotypes. This is important for the generation of extracellular matrix (ECM), which is eventually deposited in the intima [17]. Eventually a permanent matrix of collagen type I and III is produced and this allows the complete healing of the wound. Much of the information regarding the interactions between stents and arterial walls has come from the cardiology literature and it is yet to be determined if the same processes occur in the intracranial arteries treated with flow-diverting stents.

Histological studies have been conducted to determine how stents repair aneurysms. Kadirvel et al. [18] recently showed that, in rabbits, the formation of this neointima begins rapidly after stent deployment and that the smooth muscle cells grow over the stent struts that directly overlie the arterial wall. This process then continues over the aneurysmal neck until the aneurysm is excluded and the parent vessel is “reconstructed”. The authors suggest that since the smooth muscle and endothelial cells that grow over the stent struts are derived from the underlying parent artery, then optimal apposition of the flow diverter may be necessary for ideal healing. It is also possible devices with more wires, and hence more contact with the parent artery may result in a similar, but more florid response. This would theoretically not be the same with telescoped flow diverters since only the first stent is in direct contact with the vessel wall. In the study by de Vries at al. [19], using the Surpass stent (which can have up to 96 wires aimed at uniform stent pore configuration across different vessel diameters), only 4 (*n* = 30) patients had narrowing and in all these patients it was <20%. Unfortunately it was not documented which size of Surpass was used in these cases so no meaningful conclusions can be drawn from this. Interestingly, in our series only a single case treated with multiple telescoped stents developed transient in-stent stenosis.

To our knowledge there is no data currently that suggest malapposition between a flow diverter and the vessel wall has an effect on in stent stenosis although there is evidence to suggest that malapposition is associated with late stent thrombus in coronary vessels [20]. Similarly late thrombosis as a consequence of poor apposition can increase the risk for complications secondary to thromboembolic events [21, 22].

It is also known that malapposition can result in an endoleak and incomplete aneurysm occlusion [23, 24]. Two recent advances in technology may allow us to further delineate the potential role malapposition has on the incidence of in-stent stenosis. Intravascular optical coherence tomography (OCT) is based on interferometry to scan the backscatter of a near infrared light source with low coherence length that is relayed and received via a single fiberoptic wire. The wire is simultaneously rotated and pulled back to obtain a series of images at 100–200 um intervals. Alternatively, and more readily available, is high-resolution contrast-enhanced cone beam CT (Vaso-CT). This can be used to assess stent wall apposition and offers inherent advantages over standard DSA because of its 3D images as well as over MRI and CT because of the increase in spatial resolution. Flood et al. [25] compared Vaso-CT to standard DSA acquisitions and found a good correlation between the degree of stenosis measured by the two techniques. Similarly, they showed a good correlation between the Vaso-CT findings and histological examination. Importantly, Vaso-CT can prove extremely useful in the detection of eccentric stenosis where DSA can easily overlook this if the projection angle is not optimized. More recently, van der Marel et al. [26] compared Vaso-CT and OCT and they found good correlation between the findings on OCT and Vaso-CT with OCT being able to detect malapposition with 98% sensitivity and 81% specificity. Although OCT is currently not in clinical practice, these preliminary studies suggest that OCT may be an important tool to assess stent wall apposition in the future.

Further research is required on the exact determinants of in stent stenosis and whether there is a significant effect from aspects such as under or oversizing the device [27], the effect of wall shear stress (WSS) [28] or even the stent alloys [29].

Our study has several limitations inherent to a retrospective design.

## Conclusion

Any degree of ISS occurs in approximately 29% of all patients treated with a p64. More than 50% lumen loss is encountered in only 11.2%. There is a general trend for improvement over time without treatment other than continued dual antiaggregation. We would, however, advise close monitoring of these patients as ISS might progress to a critical level, eventually prompting balloon dilatation, which can be performed with a high success rate and good safety margins.

## References

[1] Saatci I, Yavuz K, Ozer C, Geyik S, Cekirge HS (2012). Treatment of intracranial aneurysms using the pipeline flow-diverter embolization device: a single-center experience with long-term follow-up results. AJNR Am J Neuroradiol.

[2] Pistocchi S, Blanc R, Bartolini B, Piotin M (2012). Flow diverters at and beyond the level of the circle of willis for the treatment of intracranial aneurysms. Stroke.

[3] Chalouhi N, Tjoumakaris S, Starke RM, Gonzalez LF, Randazzo C, Hasan D, McMahon JF, Singhal S, Moukarzel LA, Dumont AS, Rosenwasser R, Jabbour P (2013). Comparison of flow diversion and coiling in large unruptured intracranial saccular aneurysms. Stroke.

[4] Chalouhi N, Starke RM, Yang S, Bovenzi CD, Tjoumakaris S, Hasan D, Gonzalez LF, Rosenwasser R, Jabbour P (2014). Extending the indications of flow diversion to small, unruptured, saccular aneurysms of the anterior circulation. Stroke.

[5] Tomasello A, Romero N, Aixut S, Miquel MA, Macho JM, Castaño C, Coscojuela P, Lemus M, Aja L, San Roman L, Blasco J, Rovira A (2016). Endovascular treatment of intracraneal aneurysm with pipeline embolization device: experience in four centres in Barcelona. Neurol Res.

[6] Brinjikji W, Murad MH, Lanzino G, Cloft HJ, Kallmes DF (2013). Endovascular treatment of intracranial aneurysms with flow diverters: a meta-analysis. Stroke.

[7] Wang CB, Shi WW, Zhang GX, Lu HC, Ma J (2016). Flow diverter treatment of posterior circulation aneurysms. A meta-analysis. Neuroradiology.

[8] Cohen JE, Gomori JM, Moscovici S, Leker RR, Itshayek E (2014). Delayed complications after flow-diverter stenting: reactive in-stent stenosis and creeping stents. J Clin Neurosci.

[9] John S, Bain MD, Hui FK, Hussain MS, Masaryk TJ, Rasmussen PA, Toth G (2016). Long-term follow-up of in-stent stenosis after pipeline flow diversion treatment of Intracranial aneurysms. Neurosurgery.

[10] Lubicz B, Van der Elst O, Collignon L, Mine B, Alghamdi F (2015). Silk flow-diverter stent for the treatment of intracranial aneurysms: a series of 58 patients with emphasis on long-term results. AJNR Am J Neuroradiol.

[11] Mitra AK, Agrawal DK (2006). In stent restenosis: bane of the stent era. J Clin Pathol.

[12] Grewe PH, Deneke T, Machraoui A, Barmeyer J, Müller KM (2000). Acute and chronic tissue response to coronary stent implantation: pathologic findings in human specimen. J Am Coll Cardiol.

[13] Inoue T, Croce K, Morooka T, Sakuma M, Node K, Simon DI (2011). Vascular inflammation and repair: implications for re-endothelialization, restenosis, and stent thrombosis. JACC Cardiovasc Interv.

[14] Welt FG, Tso C, Edelman ER, Kjelsberg MA, Paolini JF, Seifert P, Rogers C (2003). Leukocyte recruitment and expression of chemokines following different forms of vascular injury. Vasc Med.

[15] Otsuka F, Finn AV, Yazdani SK, Nakano M, Kolodgie FD, Virmani R (2012). The importance of the endothelium in atherothrombosis and coronary stenting. Nat Rev Cardiol.

[16] Chaabane C, Otsuka F, Virmani R, Bochaton-Piallat ML (2013). Biological responses in stented arteries. Cardiovasc Res.

[17] Campbell JH, Campbell GR (2012). Smooth muscle phenotypic modulation – a personal experience. Arterioscler Thromb Vasc Biol.

[18] Kadirvel R, Ding YH, Dai D, Rezek I, Lewis DA, Kallmes DF (2014). Cellular mechanisms of aneurysm occlusion after treatment with a flow diverter. Radiology.

[19] De Vries J, Boogaarts J, Van Norden A, Wakhloo AK (2013). New generation of flow diverter (surpass) for unruptured Intracranial aneurysms: a prospective single-center study in 37 patients. Stroke.

[20] Cook S, Wenaweser P, Togni M, Billinger M, Morger C, Seiler C, Vogel R, Hess O, Meier B, Windecker S (2007). Incomplete stent apposition and very late stent thrombosis after drug-eluting stent implantation. Circulation.

[21] Klisch J, Turk A, Turner R, Woo HH, Fiorella D (2011). Very late thrombosis of flow-diverting constructs after the treatment of large fusiform posterior circulation aneurysms. AJNR Am J Neuroradiol.

[22] Pierot L, Wakhloo AK (2013). Endovascular treatment of intracranial aneurysms: current status. Stroke.

[23] Carneiro A, Rane N, Küker W, Cellerini M, Corkill R, Byrne JV (2014). Volume changes of extremely large and giant intracranial aneurysms after treatment with flow diverter stents. Neuroradiology.

[24] Mattingly T, Van Adel B, Dyer E, Lopez-Ojeda P, Pelz DM, Lownie SP, Marotta T, Boulton M (2015). Failure of aneurysm occlusion by flow diverter: a role for surgical bypass and parent artery occlusion. J Neurointerv Surg.

[25] Flood TF, van der Bom IM, Strittmatter L, Puri AS, Hendricks GM, Wakhloo AK, Gounis MJ (2015). Quantitative analysis of high-resolution, contrast-enhanced, cone-beam CT for the detection of intracranial in-stent hyperplasia. J Neurointerv Surg.

[26] van der Marel K, Gounis MJ, Weaver JP, de Korte AM, King RM, Arends JM, Brooks OW, Wakhloo AK, Puri AS (2016). Grading of regional apposition after flow-diverter treatment (GRAFT): a comparative evaluation of vasoCT and intravascular OCT. J Neurointerv Surg.

[27] Berg P, Iosif C, Ponsonnard S, Yardin C, Janiga G, Mounayer C (2016). Endothelialization of over- and undersized flow-diverter stents at covered vessel side branches: an in vivo and in silico study. J Biomech.

[28] Benard N, Coisne D, Donal E, Perrault R (2003). Experimental study of laminar blood flow through an artery treated by a stent implantation: characterisation of intra-stent wall shear stress. J Biomech.

[29] Adam Z, Turley A, Mason JM, Kasim AS, Newby D, Mills N, Padfield G, Thompson L, Morley R, Hall JA, Wright RA, Muir DF, Sutton AG, Swanson N, Carter J, Bilous R, Jones S, de Belder MA (2016). The SSTARS (STeroids and Stents Against Re-Stenosis) trial: different stent alloys and the use of peri-procedural oral corticosteroids to prevent in-segment restenosis after percutaneous coronary intervention. Int J Cardiol.

